# Endothelin-1 promotes hypertrophic remodelling of cardiac myocytes by activating sustained signalling and transcription downstream of endothelin type A receptors

**DOI:** 10.1016/j.cellsig.2017.04.010

**Published:** 2017-08

**Authors:** Caroline R. Archer, Emma L. Robinson, Faye M. Drawnel, H. Llewelyn Roderick

**Affiliations:** a Laboratory of Experimental Cardiology, Dept. of Cardiovascular Sciences, KU Leuven, Campus Gasthuisberg, Herestraat 49, B-3000, Leuven, Belgium; b The Babraham Institute, Babraham, Cambridge, CB22 3AT, UK

**Keywords:** Endothelin-1, Cardiac hypertrophy, Signalling, MAPK, GPCRs, Act D, actinomycin D, Ang II, angiotensin II, ANF, atrial natriuretic factor, BNP, brain natriuretic factor, CN, calcineurin, CVD, cardiovascular disease, ara-C, cytosine b-d-arabinofuranoside, DPM, disintegrations per minute, DN β-Arr1, dominant negative β-arrestin-1, DUSP6, dual specificity phosphatase-6, ET-1, endothelin-1, ECE, endothelin converting enzyme, ET_A_ receptor, endothelin type A receptor, ET_B_ receptor, endothelin type B receptor, ECC, excitation contraction coupling, ERK1/2, extracellular signal-regulated kinase 1/2, GPCR, G-protein coupled receptor, IEG, immediate early gene, MAPK, mitogen activated protein kinase, MEF2, myocyte enhancer factor-2, α-MHC, myosin heavy chain, α isoform, β-MHC, myosin heavy chain, β isoform, NRVMs, neonatal rat ventricular myocytes, NFAT, nuclear factor of activated T cells, PE, phenylepherine, PD, PD184352, SRF, serum response factor, TAC, transverse aortic constriction, TCA, trichloroacetic acid

## Abstract

G-protein coupled receptor (GPCR) mediated activation of the MAPK signalling cascade is a key pathway in the induction of hypertrophic remodelling of the heart – a response to pathological cues including hypertension and myocardial infarction. While levels of pro-hypertrophic hormone agonists of GPCRs increase during periods of greater workload to enhance cardiac output, hypertrophy does not necessarily result. Here we investigated the relationship between the duration of exposure to the pro-hypertrophic GPCR agonist endothelin-1 (ET-1) and the induction of hypertrophic remodelling in neonatal rat ventricular myocytes (NRVM) and in the adult rat heart *in vivo*. Notably, a 15 min pulse of ET-1 was sufficient to induce markers of hypertrophy that were present when measured at 24 h *in vivo* and 48 h *in vitro*. The persistence of ET-1 action was insensitive to ET type A receptor (ET_A_ receptor) antagonism with BQ123. The extended effects of ET-1 were dependent upon sustained MAPK signalling and involved persistent transcription. Inhibitors of endocytosis however conferred sensitivity upon the hypertrophic response to BQ123, suggesting that endocytosis of ET_A_ receptors following ligand binding preserves their active state by protection against antagonist. Contrastingly, α_1_ adrenergic-induced hypertrophic responses required the continued presence of agonist and were sensitive to antagonist. These studies shed new light on strategies to pharmacologically intervene in the action of different pro-hypertrophic mediators.

## Highlights

• Acute ET-1 exposure elicits a long-lasting cardiac myocyte hypertrophic response.
• ET-1 effects depend on persistent MAPK signalling and active transcription.
• ET-1 elicited hypertrophy is insensitive to subsequent ET_A_ receptor antagonism.
• Endocytosis inhibition potentiates ET-1-induction of hypertrophy markers.
• Endocytosis inhibition sensitises effects of ET-1 to ET_A_ receptor antagonist.

## 1 Introduction

Cardiovascular diseases (CVD) are a leading cause of mortality, accounting for 40% of deaths in males and 49% of deaths in females in Europe [1]. CVDs comprise a diverse group of pathologies, including hypertension, myocardial infarction and cardiomyopathy that impact upon the function of the heart. Through increased resistance of the vasculature, loss of functional myocardium or poorly functioning contractile machinery, these conditions elevate the workload on the heart resulting in hypertrophic remodelling, whereby the heart increases in size without myocyte proliferation [2]. Cardiac remodelling subsequent to pathological cues, often associated with increased afterload, is maladaptive. It is characterised by an increase in wall thickness without an increase in chamber volume (concentric hypertrophy) and is a prequel to heart failure. At the level of the cardiomyocyte, hypertrophy involves enhanced protein synthesis and an increase in sarcomere number and organization [3], [4]. Cardiac hypertrophy also involves substantial transcriptional remodelling. Most notably, pathological hypertrophy is characterised by a re-expression of a foetal gene programme that includes natriuretic peptides, ANF (*NPPA*) and BNP (*NPPB*) and a decrease in expression of the adult α isoform of myosin heavy chain α-MHC (*MYH6*) relative to the foetal isoform, β-myosin heavy chain; β-MHC (*MYH7*). Physiological hypertrophy however does not lead to foetal gene programme induction and involves an increase in α-MHC (*MYH6*) relative to β-MHC (*MYH7*) [4].

While the initial stages of pathological hypertrophy are often adaptive, continued stress leads to decompensation of the response culminating in heart failure [3]. Indeed, while chronic application of the adrenergic agonist dobutamine induces a maladaptive hypertrophic response, when applied in a pulsatile manner, the hypertrophic response is adaptive and without fibrosis [5]. A key distinguishing feature of pathological hypertrophic responses is the primary involvement of G_q_-protein coupled receptor (GPCR) signalling [4], [5], [6], [7]. GPCR agonists including endothelin-1 (ET-1) and Angiotensin II (Ang II) are chronically elevated in disease, including in the myocardium [8], [9]. Acting *via* cognate receptors on cardiac myocytes, they elicit acute effects on physiology, altering excitation contraction coupling (ECC) and more chronic effects on cardiac growth through altered transcription [10], [11], [12]. Pathology-inducing neurohormones may also be elevated transiently during acute periods of stressful activity such as the fight/flight response but do not necessarily induce long lasting phenotypic responses [13], [14]. Indeed acutely, epinephrine and norepinephrine induce immediate chronotropic and inotropic responses, whereas chronic elevation in the levels of these hormones is required to stimulate hypertrophic remodelling [13], [14].

GPCR activation results in the engagement of multiple downstream signalling cascades [6]. Notable amongst these in the regulation of cardiac hypertrophy is the extracellular signal-regulated kinase 1/2 (ERK1/2) MAPK pathway. This signalling cascade plays a central role in hypertrophy induction through the stimulation of expression and phosphorylation of immediate early gene (IEG) and activation of transcription factors such as serum response factor (SRF) and myocyte enhancer factor-2 (MEF2). Indeed, the expression of the majority of genes induced following ET-1 is sensitive to MAPK inhibition, indicating the critical role of this pathway in signalling cardiac remodelling [15]. ERK1/2 activation is detected within minutes of cell exposure to agonist or mechanical stretch *in vitro* [16], [17], [18] and within 15 min of transverse aortic constriction (TAC) *in vivo* [19], [20]. Downstream consequences of this signalling activity are also detected within minutes *in vitro*, as MAPK-stimulated IEG expression is detected within 15 min of exposure to hypertrophic stimuli [17], [18], [21]. Consistent with their role as transcription factors, the induced IEGs stimulate the expression of multiple downstream targets, including transcription factors, which then promote the expression of further downstream targets. Indeed, distinct gene populations are sequentially expressed following ET-1 stimulation in NRVMs [15], [21]. This would suggest that, following the activation of expression of the first phase of genes, a cascade is set in motion that would lead to the induction of subsequent waves of gene expression [20], [22]. While the activity of multiple signalling pathways required for hypertrophy is rapidly increased proximal to hypertrophic stimulus, whether the activity of these signalling pathways is also required for later stages in evolution of the hypertrophic response has not been determined [10], [19], [22], [23].

While these observations would suggest that sustained signalling activity is required to elicit the effects of GPCR stimulation, signalling pathways including MAPK, exhibit rapid and transient increases in their activity following receptor engagement that peaks in minutes and decays to baseline within 4 h [15], [20]. The maximal induction of IEG genes also peaks in a relatively short time period of 1 h, after which their expression decays. The transient nature of these responses is a product of efficient negative feedback mechanisms that act at multiple levels downstream of GPCR activation [6].

The rapid, yet transient activation of signalling pathways following GPCR engagement, relative to the phenotypic outcome of their activation, raises the question of how these apparently temporally dissociated processes are coupled. In particular it has not yet been determined whether the peak signalling activity observed following GPCR engagement is sufficient to induce hypertrophy to its full extent, or whether signalling beyond this initial burst of activity is required [22], [23]. A second elevation in activity of p38 and ERK1/2 7 days after pressure overload induced by aortic constriction in mice has however been observed, suggesting that the role of signalling pathways in hypertrophy induction goes beyond that proximal to the initial stimulus [20]. Here we tested the hypothesis that sustained activity of signalling pathways is required for induction of gene expression associated with pathological hypertrophy. We investigated the time of onset and duration of signalling activity required for the induction of cardiomyocyte hypertrophy. As a paradigm of hypertrophic signalling, we focussed at the level of the ET_A_ receptor, its activation of the MAPK signalling cascade and the myocyte hypertrophic response. Using primary cultures of ventricular myocytes as a tractable model that allowed the data sampling required to obtain insights into these early events, we determined that potent pro-hypertrophic effects of ET-1 (when measured at up to 24 h) are engaged within the first 15 min of its application. Notably, *in vivo* as *in vitro*, 15 min infusion of ET-1 was sufficient to elicit a hypertrophic response 24 h later. However, persistent signalling after 15 min ET-1 application was required for expression of cellular indices of hypertrophy including increased protein synthesis and ANF mRNA and protein expression. Notably, the persistent signalling from ET_A_ receptors was insensitive to antagonist in a manner that was modified by inhibition of endocytosis. These effects of ET_A_ receptors contrasted with that of the α1 adrenergic receptor, which required the continued presence of agonist for the induced-phenotype to be maintained.

## 2 Materials and methods

### 2.1 Materials

Chemicals, buffers and tissue culture reagents were from Sigma-Aldrich or Thermo Fisher Scientific, unless noted otherwise. Sources of other reagents are specified.

### 2.2 NRVM isolation and culture

Animal experiments were performed in accordance with the guidelines from the Home Office code of practice for humane killing of animals under Schedule 1 of the Animals (Scientific Procedures) Act 1986 (UK) and in accordance with the European Directive 2010/63/EU and approved by the Ethical Committee for Animal Experiments of the KU Leuven (Belgium). Neonatal rat ventricular myocytes (NRVMs) were isolated from 2 to 3 day old Wistar rats (Charles River) as described previously [24]. Based on α-actinin staining, NRVM cultures were deemed > 95% pure. After a pre-plating step to remove adherent non-myocytes, myocytes were seeded in plating medium (4 parts DMEM with 4.5 g/l d-glucose, 585 mg/l l-glutamine, 110 mg/l sodium pyruvate and 1 part M199, supplemented with 10% horse serum, 5% foetal calf serum, 1 mM sodium pyruvate, 1 mM MEM non-essential amino acids, 1% antibiotic/antimycotic and 3 μM cytosine b-d-arabinofuranoside; ara-C) either on 1% gelatin-coated dishes or 25 μg/ml laminin-coated coverslips. Cells were washed and left in plating medium for a further 24 h. NRVMs were serum-starved in maintenance medium (4 parts DMEM with 4.5 g/l d-glucose, 585 mg/l l-glutamine, 110 mg/l sodium pyruvate and 1 part M199 supplemented with 1 mM sodium pyruvate, 1% antibiotic/antimycotic, 3 μM ara-C and 5 ng/ml sodium selenite) for 24 h before commencing treatments. All agonists and antagonists and their vehicle controls were diluted in maintenance media for the durations stated. Where agonists were applied together with agonist, NRVMs were exposed to antagonist 60 min prior to agonist addition.

### 2.3 Cell treatments

ET-1 was from Merck Millipore, prepared as a 200 μM stock in 5% acetic acid and applied at 100 nM. Phenylephrine (PE) was from Sigma-Aldrich, prepared as a 10 mM stock in water and applied at 10 μM. Inhibitor or vehicle was applied 30 min prior to cell stimulation with agonist. BQ123 was from Merck Millipore, prepared in water and was used at 1 μM. Prazosin was from Tocris, prepared in DMSO and was applied at 100 nM. Dynole (Dynole 34-2) was from Tocris, was prepared in Ethanol and was applied at 10 μM. PD184352 (PD) was from Sigma-Aldrich, prepared in DMSO and used at 10 μM. Actinomycin D (Act D) was from Tocris, prepared in DMSO and applied at 2 μg/ml. Controls were exposed to vehicle.

### 2.4 *In vivo* ET-1 infusion

250–300 g Wistar male rats were obtained from Harlan (NL). Anesthesia was induced using ketamine and xylazine in combination (100 mg/kg ketamine, 10 mg/kg xylazine) by intraperitoneal injection. During anesthesia and throughout the procedure, body temperature was maintained with a heated mat (Sanitas). After exposure of the jugular vein, a 30 gauge needle attached to a catheter was introduced. 1 μg/kg of ET-1 was then administered over a 15 min period. This dose has previously been shown to induce a cardiac effect with minimal effects on blood pressure [25], [26]. For administration, ET-1 was diluted to 1 ng/μl in saline and infused at a rate of 20 μl/min (total volume 300 μl) using a digital pump dispenser (Harvard). At this rate, acute vasoconstrictive effect of a single rapid injection of the same dosage are minimised [27]. Control rats were infused with saline. After the 15 min infusion, one group of control and ET-1 treated rats were immediately sacrificed by cervical dislocation. For a second group of saline and ET-1 infused rats, the cannula was removed, wound sutured and the animals allowed to recover for 24 h after which they were sacrificed by cervical dislocation following anesthesia with isoflurane. In all cases, hearts were removed at point of sacrifice, rinsed in saline to remove excess blood and then snap frozen in liquid nitrogen.

### 2.5 Generation of adenoviral constructs

The GFP adenovirus was as previously described [10]. The GFP-tagged, dominant negative β-Arrestin-1 (DN β-Arr1) was kindly provided as cosmid by Prof. Antonio Porcellini (Università degli Studi del Molise, Italy) [28]. The DN β-Arr1 adenovirus was generated following transfection into HEK293 cells. After amplification in HEK293 cells, virus was purified using the Vivapure AdenoPack 100 viral purification kit (Sartorius). Viral titer was determined by end-point dilution and used at a concentration that transduced > 95% of cells. For infection, virus was added in a minimal volume of media overlying the cells and incubated for 3 h. Virus-containing media was then removed and either replaced with media or media-containing agonist [10].

### 2.6 Immunofluorescence

NRVMs were washed three times in PBS and then immersed in fixation buffer (2% paraformaldehyde (w/v), 0.05% glutaraldehyde (v/v) in PBS) at room temperature for 15 min. Cells were washed a further three times with PBS to ensure removal of fixation buffer and permeabilised in PBS containing 0.2% Triton X-100 for 15 min at room temperature. After removal of permeabilisation buffer, cells were incubated with blocking buffer (0.1% Triton X-100, 5% goat serum in PBS) for 1 h to block non-specific protein binding sites. NRVMs were then exposed to primary antibodies - polyclonal anti-ANF (1:500; Bachem Cat# T-4014.0400, RRID:AB_518074), mouse monoclonal anti-α-actinin (1:300; Sigma-Aldrich Cat# A7811, RRID:AB_476766) or mouse monoclonal anti-β-arrestin1 (1:100; BD Biosciences Cat# 610550, RRID:AB_397907) diluted in blocking buffer and incubated for 1 h. Excess antibodies were removed by 4 exchanges of PBS containing 0.1% Triton X-100 over a period of 1 h. Primary Antibodies were detected with Alexa Fluor® conjugated secondary antibodies (Thermo Fisher Scientific), diluted at 1:500 in PBS containing 0.1% Triton X-100 and 2% goat serum, which was left on the cells for 1 h. Cells were then washed every 15 min for an hour followed by a PBS wash, which was repeated 3 times. After removal of PBS, cells were mounted by inverting onto Vectashield mounting medium containing DAPI (Vector labs), which served to counterstain nuclei. Slides were imaged using an Olympus FV1000 confocal microscope attached to an Olympus IX81, equipped with a 40X/1.3 NA UPlanFI oil immersion objective. Immunolabelling of samples was detected by confocal microscopy following excitation with laser lines at 405 nm (DAPI, nuclei), 488 nm (Alexa Fluor® 488; α-actinin) and (Alexa Fluor® 568; ANF or β-arrestin1) 543 nm. For quantification of ANF, the percentage of myocytes with perinuclear ANF in the field of view was determined [10], [29]. For each sample, four non-overlapping areas of the cover slips were counted, which totalled > 350 cells per cover slip enumerated.

### 2.7 Cell surface area analysis

Cell planimetry was carried out as previously described with some modifications [10]. NRVMs plated on gelatin-coated 96 well-dishes and exposed to conditions described were stained with antibodies against α-actinin according to the immunofluorescence protocol described above. After the washes to remove excess secondary antibody, NRVMs were stained with Hoechst 33342 (Thermo Fisher Scientific; 10 mg/ml) diluted in PBS (1:10,000) for 20 min. NRVMs were washed a further 3 times in PBS and left in a final volume of 100 μl PBS for imaging. Cells were imaged using a BD Pathway 855 high-content imaging system. This system was configured with an Olympus 40 ×/0.75 NA UPlan FLN air objective and a Hamamatsu ORCA ER cooled CCD camera. Caliper software (BD pathway software) was used for image acquisition, whereby a 5 × 5 montage of each well of cells was acquired and specified using a pre-programmed macro. Cell boundaries were manually demarcated and area determined using ImageJ software (NIH). Duplicates of each treatment were analysed with no < 200 cells measured per condition.

### 2.8 Protein synthesis measurement by ^3^H Leucine incorporation

Incorporation of ^3^H Leucine into newly synthesised proteins is a robust method to assess the increased protein synthesis associated with hypertrophy [30]. During the course of treatments with agonists/inhibitors, 0.5 μCi/ml [^3^H] Leucine (Perkin Elmer) was added to the maintenance medium. Following treatments, cells were washed twice with ice cold PBS. After washing, proteins were precipitated with 5% trichloroacetic acid (TCA) 1 h on ice. TCA was removed by washing twice with ice cold PBS and the precipitated proteins were solubilised by incubating cells with 1 N NaOH for 1 h at room temperature. Following protein solubilisation, the sample was transferred to scintillation vials containing 5 ml Ultima Gold Scintillation Cocktail (Perkin Elmer). Tubes were capped, inverted 4 times and then incubated at room temperature for 10 min to ensure complete mixing. Disintegrations per minute (DPM) were counted for 2 min per sample in a scintillation counter (Packard Tricarb 4000).

### 2.9 Quantitative RT-PCR

Total RNA was isolated using Qiazol (Qiagen) or Tri Reagent (Sigma-Aldrich) followed by chloroform extraction according to the manufacturer's instructions, with the addition of 1 μl glycogen in the final ethanol wash step as a visible inert carrier of RNA. RNA was reverse transcribed with Superscript II Reverse Transcriptase (Thermo Fisher Scientific). For isolation of RNA from tissue, ~ 1 mm^3^ left ventricle was thawed in TRI Reagent (SIGMA) and then homogenized using a FastPrep®-24 Instrument and Lysing Matrix D in 2 ml Tubes (MP Biomedicals). RNA was chloroform-extracted according to manufacturer's instruction and isopropanol-precipitated with the addition of 1 μl glycogen in the final ethanol wash step as a visible inert carrier of RNA. 500 ng RNA was used for cDNA synthesis using 0.75 μl SuperScript™ II Reverse Transcriptase (Invitrogen) per sample, according to the manufacturer's protocol and diluted 1:20 with molecular biology grade water (Ambion).

Primers for Housekeeping genes and hypertrophic markers spanning exon boundaries were designed previously [10] (Supplementary Table 1). Primers to amplify nascent RNA transcripts were designed against the intronic regions of *Nppa* and *c*-*Fos* (Supplementary Table 1). Reactions were performed using Platinum SYBR Green Supermix (Thermo Fisher Scientific) or using GoTaq qPCR Mastermix (Promega) on a Chromo4 Real-time PCR Detector (Bio-Rad) or Light Cycler 480 (Roche). The mean C_T_ values were converted into quantities relative to the control sample known as the comparative ∆ C_T_ method. The indices for hypertrophy were then normalised to the geometric mean of the abundance of the 3 most stable housekeeping genes from 14-3-3 protein zeta/delta (*Ywhaz*), TATA box binding protein (*Tbp*), Glyceraldehyde 3-phosphate dehydrogenase (*Gapdh*), Troponin T (*Tnnt2*) and β-2 microglobulin (*B2m*) across the conditions used were selected using the GeneNorm method [10], [31]. For all figures other than Fig. 6, *Gapdh*, *Tnnt2* and *B2m* were used. The quantitation method used generated a normalised value for the fold change in expression of the hypertrophic gene marker.

### 2.10 Immunoblotting

Immunodetection of proteins following SDS-PAGE and western blotting (immunoblotting) was carried out as previously described, with minor modifications [24], [32]. NRVMs were scraped into RIPA buffer (10 mM Tris-HCL, pH 7.5, 140 mM NaCl, 1 mM EDTA, 0.5 mM EGTA, 1% Triton X-100, 0.1% SDS, 0.1% sodium deoxycholate, 1 × protease inhibitor cocktail (Sigma-Aldrich), 1 × phosphatase inhibitor cocktail (Sigma-Aldrich), transferred to chilled 1.5 ml tubes and incubated on ice for 30 min. After this period, cell debris was removed by centrifugation at 15,000*g* for 5 min at 4 °C. The protein concentration of the lysate was determined by BCA assay (Thermo Fisher Scientific). 10–20 μg of protein per sample was resolved on a 4–12% gradient NuPAGE Bis-Tris SDS gel (Thermo Fisher Scientific). Proteins were transferred to Immobilon-P PVDF membrane (Merck Millipore) or 0.1 μ nitrocellulose (GE Healthcare) after which non-specific protein binding sites were blocked by incubation in Tris-buffered saline containing 0.05% Tween (TBST) and 5% skimmed milk (ECL blocking buffer) for 1 h (detection by enhanced chemiluminescence; ECL detection) or in TBS blocking buffer (Licor) diluted 1:1 in TBS (for Licor-based detection). For detection of proteins of interest, membranes were incubated overnight at 4 °C with the required antibodies diluted in blocking buffer (ECL) or TBS blocking buffer (Licor) diluted 1:10 in TBS (for Licor-based detection); mouse monoclonal GAPDH (1:1000; Sigma-Aldrich Cat# G8795, RRID:AB_1078991), mouse monoclonal phospho p42/44 MAPK (1:1000; Cell Signaling Technology Cat# 9106S, RRID:AB_331768), rabbit monoclonal phospho p42/44 MAPK (1:1000; Cell Signaling Technology Cat#4377) mouse monoclonal ERK1 (1:1000; BD Biosciences Cat# 610031, RRID:AB_397448), mouse monoclonal ERK1/2 (1:1000, Cell Signaling Technology Cat # 4696) mouse monoclonal anti-β-arrestin1 (1:500; BD Biosciences Cat# 610550, RRID:AB_397907). For detection of phosphoproteins by ECL, milk in the blocking buffer was exchanged for 5% bovine serum albumin (BSA). Antibody binding was detected using horse radish peroxidase (HRP)-conjugated secondary antibodies (Jackson Immunoresearch) diluted 1:10,000 in blocking buffer, which was incubated with the membranes for 1 h on a rocking platform. Excess secondary antibody was then removed by 4 washes in TBST over 1 h, followed by a final wash with TBS. Detection of proteins was by Enhanced Chemiluminescence using Supersignal West Pico Chemiluminescent substrate (Thermo Fisher Scientific) and exposure to film. For Licor infrared imaging system-based detection, immunoreactive proteins were detected using IR800 and Alexa680 conjugated secondary antibodies, which were diluted at 1:10,000 in TBST. Immunoreactive bands were quantified using Image Studio software (Licor).

### 2.11 Statistical analysis

Statistical analyses were performed using GraphPad Prism 6. Data represents a minimum of 3 independent biological replicates, sample numbers for each experiment indicated in the figure legend. For testing statistical significance of three or more conditions, the means were compared by one-way analysis of variance (ANOVA) followed by post-hoc Holm-Sidak's Multiple Comparison test (for testing statistical significance between groups of the same dataset, illustrated with a line between groups). A one-sample *t*-test was used for testing significance between 2 experimental conditions within a data set. Data was taken as significant when p was < 0.05, which was represented as *. p-Values < 0.01, 0.001, 0.0001 were represented by **, *** and ****, respectively.

## 3 Results

### 3.1 Transient ET-1 exposure upregulated indices of hypertrophy

**Fig. 1:**
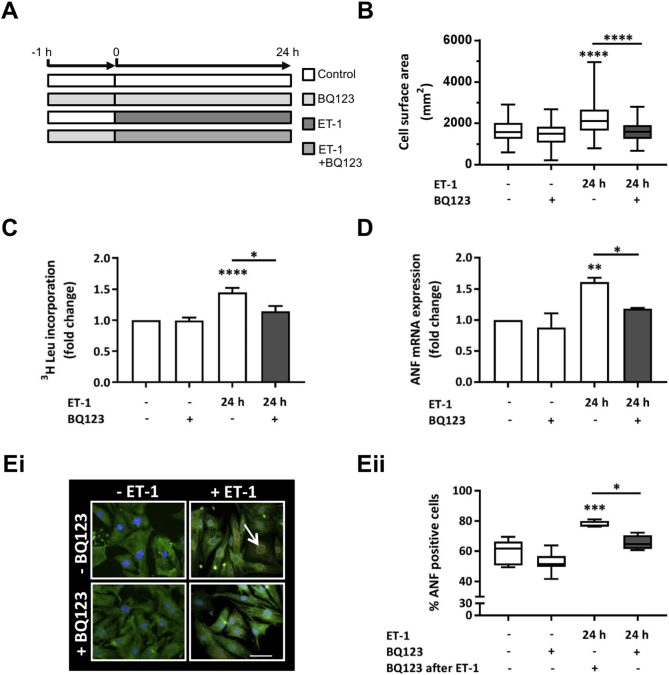
Stimulation of NRVMs with ET-1 for 24 h induces upregulation of hypertrophic markers. A. Cartoon illustrating the experimental protocol and time course of ET-1 and BQ123 application. Cells were harvested and assays were performed at 24 h after agonist addition. B. Box plots showing cell surface area measurements of NRVMs exposed to ET-1 ± BQ123 for 24 h. Box spans the 25th and 75th percentile, the line marks the median and the whiskers mark the minimum and maximum data points from at 3 primary cell preparations (n ≥ 30 cells per group, ****p < 0.0001). C. ^3^H leucine incorporation assay of protein synthesis showing fold change relative to the control upon stimulation with ET-1 ± BQ123. Each bar represents the mean ± SEM (n = 4 primary cell preparations, *p < 0.05, ****p < 0.0001). D qRT-PCR analysis of ANF mRNA abundance relative to the control upon stimulation with ET-1 ± BQ123. Each bar represents the mean ± SEM (n = 3 primary cell preparations, *p < 0.05, **p < 0.01). Ei. Representative confocal images of NRVMs exposed to ET-1 ± BQ123 for 24 h and immunostained for ANF in red, α-actinin in green and nuclei in blue (DAPI). Scale bar represents 50 μm. Peri-nuclear rings of ANF in red are indicated with a white arrow. Eii. Percentage of NRVMs treated with ET-1 ± BQ123 exhibiting a peri-nuclear ring of ANF. Box spans the 25th and 75th percentile, the line marks the median and the whiskers mark the minimum and maximum data points from 4 primary cell preparations (*p < 0.05, ***p < 0.001). Statistical analysis was performed using a one-way ANOVA followed by post-hoc Holm-Sidak's Multiple Comparison test (symbols above bars/plots). (For interpretation of the references to colour in this figure legend, the reader is referred to the web version of this article.)

Primary cultures of NRVMs are an established *in vitro* model for analysis of cardiac hypertrophic signalling [33], [34], [35]. Exposure of myocytes to G_q_-coupled agonists, including ET-1, for 24 h induces a cellular hypertrophic response that exhibits features of the *in vivo* myocyte hypertrophic response, such as expression of a foetal gene programme (*e.g. Nppa* and *Nppb*; here described as ANF and BNP, respectively), increased protein synthesis and increased cell size [10], [23], [34], [35]. The induction of indices of hypertrophy in NRVMs following exposure to ET-1 (100 nM) for 24 h validated these previous findings (Fig. 1). In particular, ET-1 induced increased cell surface area and protein synthesis (Fig. 1B and C, respectively). Consistent with its use as a surrogate marker of hypertrophy increased in patients with cardiac disease and in animal models of hypertrophy [36], [37], the expression of ANF mRNA and protein was also markedly upregulated following ET-1 exposure (Fig. 1D, Ei and Eii). All of these effects of ET-1 upon NRVMs were inhibited by application of the ET_A_ receptor antagonist, BQ123 (1 μM), which was applied 60 min prior to and during ET-1. BQ123 had no affect when applied alone (Fig. 1B–E).

**Fig. 2:**
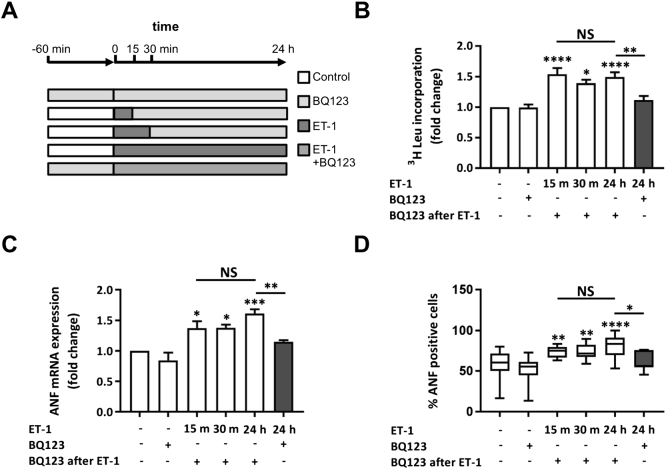
ET-1 application for 15 min with ET_A_ receptor antagonism upon removal upregulates hypertrophic markers in NRVMs when measured at 24 h. A. Cartoon illustrating the experimental protocol and time course of ET-1 and BQ123 application. Cells were harvested and assays were performed at 24 h after agonist addition. B. ^3^H leucine incorporation assay of protein synthesis showing fold change relative to the control upon stimulation with ET-1 ± BQ123. Each bar represents the mean ± SEM (n = 3 primary cell preparations, *p < 0.05, **p < 0.01, ****p < 0.0001, NS; not significant). C. qRT-PCR analysis of ANF mRNA abundance relative to the control upon stimulation with ET-1 ± BQ123. Each bar represents the mean ± SEM (n = 4 primary cell preparations, *p < 0.05, **p < 0.01, ***p < 0.001, NS; not significant). D. Percentage of NRVMs treated with ET-1 ± BQ123 exhibiting a peri-nuclear ring of ANF. Box spans the 25th and 75th percentile, the line marks the median and the whiskers mark the minimum and maximum data points from 8 primary cell preparations (*p < 0.05, **p < 0.01, ****p < 0.0001, NS; not significant). Statistical analysis was performed using a one-way ANOVA followed by post-hoc Holm-Sidak's Multiple Comparison test (symbols above bars/plots).

Having shown the pro-hypertrophic effect of ET-1 and its sensitivity to BQ123, we set out to determine the minimum duration of ET-1 exposure required to induce the hypertrophic phenotype observed at 24 h. ET-1 was applied to NRVMs for 15 or 30 min, at which time it was removed by 2 washes with fresh medium and BQ123 added to prevent further signalling from the ET_A_ receptor (Fig. 2A). NRVMs were harvested for analysis 24 h from the point of agonist addition. An increase in general protein synthesis and of ANF mRNA and protein expression was observed for each of the ET-1 exposure times analysed (Fig. 2B, C and D, respectively). An increase in BNP mRNA, which is also typically observed in the hypertrophic response [38], was detected in NRVMs exposed to ET-1 for 15 or 30 min (Supplementary Fig. 1). Notably, the magnitude of hypertrophic response (measured at 24 h) was no different between NRVMs exposed to ET-1 for 15 min or 24 h.

**Fig. 3:**
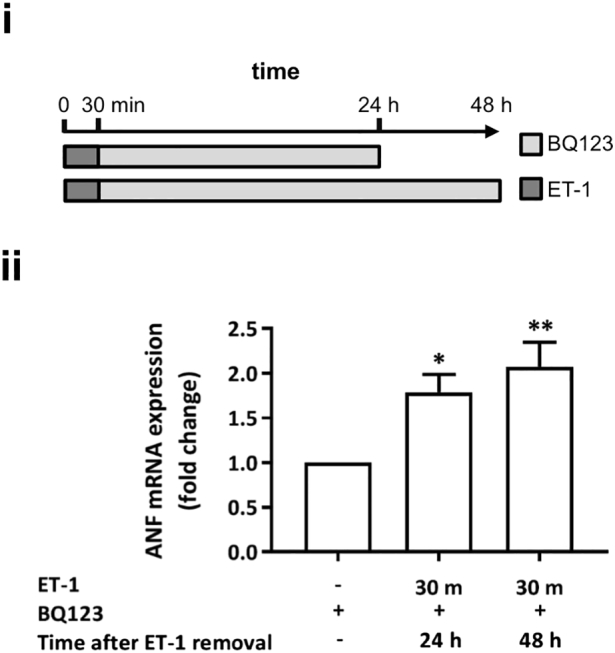
ET-1 application for 30 min with ET_A_ receptor antagonism upon removal upregulates hypertrophic markers in NRVMs when measured over a prolonged timecourse for up to 48 h. i. Cartoon illustrating the experimental protocol and time course of ET-1 and BQ123 application. Assays were performed at 24 or 48 h. ii. qRT-PCR analysis of ANF mRNA abundance relative to the control upon stimulation with ET-1 for 30 min + BQ123 at 24 or 48 h. Each bar represents the mean ± SEM (n = 3 primary cell preparations, *p < 0.05, **p < 0.01). Statistical analysis performed using the one-way ANOVA followed by post-hoc Holm-Sidak's Multiple Comparison (symbols above bars/plots).

To further probe the persistence of the effect of 30 min exposure to ET-1 upon the hypertrophic response and to gain closer insight into the longer time scales over which hypertrophy is induced *in vivo*, the time point for measurement of hypertrophy following the 30 min exposure to ET-1 and its replacement with BQ123 was extended to 48 h (Fig. 3). Under these conditions, the hypertrophic response was maintained up until the 48 h time point, with no diminution of the magnitude of the response observed when compared to that measured at 24 h. In addition, upregulation of ANF mRNA expression was sustained when ET-1 was incubated for 24 h and assayed 12 and 24 h later (Supplementary Fig. 2).

**Fig. 4:**
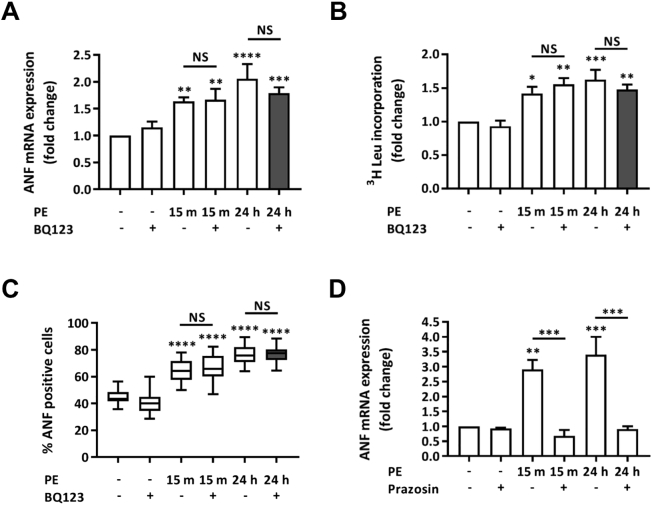
PE application for 15 min with ET_A_ receptor or α1-adrenergic receptor antagonism upon removal upregulates hypertrophic markers in NRVMs when measured at 24 h. A. qRT-PCR showing fold change in ANF mRNA expression relative to the control upon stimulation with PE ± BQ123. Assays were performed at 24 h. Each bar represents the mean ± SEM (n = 5 primary cell preparations, **p < 0.01, ***p < 0.001, ****p < 0.0001, NS; not significant). B. ^3^H leucine incorporation assay of protein synthesis showing fold change relative to the control upon stimulation with PE ± BQ123. Each bar represents the mean ± SEM (n = 4 primary cell preparations, *p < 0.05, **p < 0.01, ***p < 0.001, NS; not significant). C. Percentage of NRVMs treated with PE ± BQ123 exhibiting a peri-nuclear ring of ANF. Box spans the 25th and 75th percentile, the line marks the median and the whiskers mark the minimum and maximum data points from 3 primary cell preparations (****p < 0.0001, NS; not significant). D. qRT-PCR analysis of ANF mRNA abundance relative to the control upon stimulation with PE ± Prazosin. Assays were performed at 24 h. Each bar represents the mean ± SEM (n = 4 primary cell preparations, **p < 0.01, ***p < 0.001). Statistical analysis was performed using a one-way ANOVA followed by post-hoc Holm-Sidak's Multiple Comparison test (symbols above bars/plots).

To investigate whether the persistence of hypertrophic signalling was specific to the ET-1/ET_A_ receptor activation cascade or was a more general feature of hypertrophic responses to G_q_ agonists, the hypertrophic response to the G_q_ coupled α1 adrenergic receptor agonist PE, was analysed [18], [35]. To avoid possible influence of paracrine/autocrine signalling by ET-1 reported in NRVMs undergoing hypertrophy [10], BQ123 was applied to cells following removal of PE. As observed for ET-1, exposure of NRVMs to PE for 15 min (10 μM) was sufficient to induce expression of hypertrophy indices 24 h later (Fig. 4A, B and C). Application of BQ123 subsequent to the addition of PE to inhibit the potential activation of ET_A_ receptors did not affect PE-induction of hypertrophy, ruling out their contribution to signalling cascades activated by PE. In contrast, despite also acting through a GPCR pathway involving G_q_, application of the α1 adrenergic receptor antagonist Prazosin (100 nM) 15 min post PE application abrogated its pro-hypertrophic action [35]. Prazosin also inhibited the induction of hypertrophic markers when applied prior to PE addition (Fig. 4D). The contrasting effects of inhibition of ET_A_ and α1 adrenergic receptors subsequent to 15 min exposure of their cognate ligand, would suggest different signalling mechanisms for these receptors. In particular, that continuous ligand association is required for α1 adrenergic receptor-mediated induction of hypertrophic gene expression, whereas, ET-1 elicits its pro-hypertrophic effects within 15 min of application.

### 3.2 Persistence of the pro-hypertrophic effect of ET-1 requires sustained transcription post agonist removal

The presence of a hypertrophic response at 24 h, despite removal of ET-1 and application of BQ123 15 min after ET-1 addition, raised the question whether transcription of hypertrophy-related marker genes and protein synthesis observed at 24 h was induced within this initial stimulation time period. Levels of ANF mRNA and protein synthesis were therefore measured at 15 and 30 min subsequent to agonist application. When assayed at these time points, no induction in ANF mRNA or protein synthesis was observed (Supplementary Fig. 3Ai and B), whereas, consistent with experiments above, ANF mRNA and general protein synthesis were both elevated when measured 24 h post agonist application. Having observed no induction of ANF mRNA at 30 min, we also performed a more detailed analysis of ANF mRNA expression between from 1 h and 24 h post agonist application. This analysis revealed an induction of expression of ANF mRNA at 1 h that did not substantially alter throughout the time course of the experiment, suggesting that peak expression was reached (Supplementary Fig. 3Aii). To determine whether exposure to ET-1 for < 30 min was sufficient to elicit a transcriptional response in our experimental model and to control for detection of a response, the induction of the IEG *c*-*Fos* was also quantitated. Induction of IEG expression is a primary transcriptional response to mitogenic agonists, including GPCRs engagement in NRVMs [39]. As IEGs act as transcription factors, induction of IEG expression represents an initial step in the pathway to more wholesale transcriptional regulation [39]. Notably, while the induction of indices of hypertrophy were not detected within 30 min post agonist application, ET-1 application promoted a substantial increase in *c*-*Fos* mRNA by 15 min that peaked at 30 min and then declined after 1 h to an elevated plateau for the remainder of the experimental time course (Supplementary Fig. 3C). This expression profile contrasted with that observed for ANF mRNA, which upon eliciting its response to ET-1, remained at a similar level for the duration of the experimental time-course (Fig. 2 and Supplementary Fig. 3A).

**Fig. 5:**
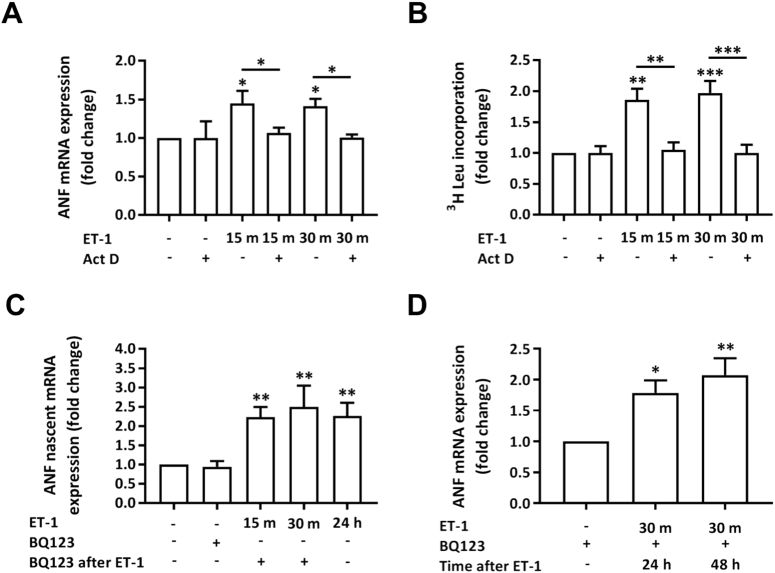
Persistence of ANF following removal of ET-1 is due to its continued active transcription. A. qRT-PCR analysis of ANF mRNA abundance relative to the control upon stimulation with ET-1 ± Act D. Assays were performed at 24 h. Each bar represents the mean ± SEM (n = 4 primary cell preparations, *p < 0.05). B. ^3^H leucine incorporation assay of protein synthesis showing fold change in protein synthesis relative to the control upon stimulation with ET-1 ± ActD. Assays were performed at 24 h. Each bar represents the mean ± SEM (n = 7 primary cell preparations, **p < 0.01, ***p < 0.001). C. qRT-PCR analysis of ANF nascent mRNA abundance relative to the control upon stimulation with ET-1 + BQ123. Assays were performed at 24 h. Each bar represents the mean ± SEM (n = 4 primary cell preparations, **p < 0.01). D. qRT-PCR analysis of ANF nascent mRNA abundance relative to the control upon stimulation with ET-1 + BQ123 at 24 or 48 h. Each bar represents the mean ± SEM (n = 3 primary cell preparations, *p < 0.05, **p < 0.01). Statistical analysis was performed using a one-way ANOVA followed by a post-hoc Holm-Sidak's Multiple Comparison test (symbols above bars/plots).

To further understand the mechanism by which hypertrophy was induced at 24 h following exposure to ET-1 for 15 min, yet absent when measured at 15 or 30 min, we tested whether the hypertrophic response at 24 h and later time points was mediated by the persistence of active transcription beyond this period of agonist exposure or was due to transcripts generated during the initial stimulation period. The requirement for persistent transcription after removal of agonist at 15, or 30 min for the hypertrophic response measured at 24 h was probed by analysing the effect of inhibition of transcript elongation by RNA polymerase II with Actinomycin D (Act D) (2 μg/ml) at the point of agonist removal [40]. As previously, ET-1 was applied to NRVMs for 15 or 30 min. At these time points, Act D or vehicle control was added in the continued presence of the hypertrophic agonist. At 24 h, the cells were assayed for ANF mRNA expression or protein synthesis. Addition of Act D 15 or 30 min post ET-1 addition inhibited the induction of ANF mRNA expression and protein synthesis normally observed (Fig. 5A and B, respectively). Since hypertrophic transcripts were not observed at the time points of Act D addition (15 and 30 min after ET-1; Supplementary Fig. 3A), the absence of these transcripts and hypertrophy at 24 h indicates that they are produced as a result of persistent transcription.

To establish whether ANF mRNA quantitated at the 24 and 48 h time points following brief agonist exposure resulted from persistent active transcription up until the point of cell harvesting, the abundance of nascent ANF transcripts at this time point was measured. Given that RNA splicing occurs concomitant with transcription [21], the analysis of nascent transcripts reflect transcriptional activity. Agonist was applied, after 15 or 30 min removed and replaced with BQ123. As for mRNA analysis, cells were harvested 24 h after the addition of agonist. A control in which agonist was applied for 24 h was also included in the analysis. Increased abundance of nascent transcripts for ANF was detected at 24 h for all conditions tested (Fig. 5C). Moreover, nascent transcripts were detected up to 48 h following a 30 min exposure to ET-1 (Fig. 5D). These findings are analogous to that observed for expression of the mature ANF transcript following ET-1 application. These data indicate that transient agonist exposure sets in motion mechanisms to induce persistent activation of the transcription machinery involved in transcribing ANF.

### 3.3 15 minute ET-1 exposure induces hypertrophy-associated transcripts at 24 h *in vivo*

**Fig. 6:**
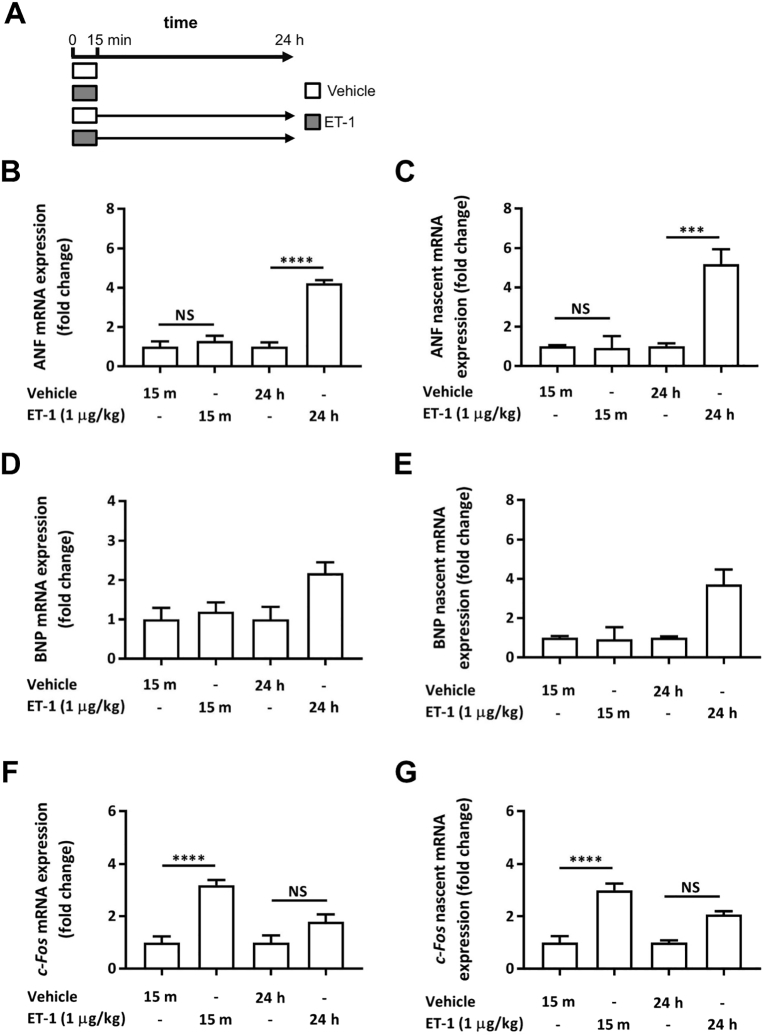
A 15 min ET-1 infusion induces hypertrophy-associated transcripts at 24 h *in vivo*. A. Cartoon illustrating the *in vivo* experimental protocol and time course of vehicle (saline) and ET-1 infusion. Vehicle control rats were infused with saline. After the 15 min infusion, one group of vehicle and ET-1 treated rats were immediately sacrificed (top bars; sample collection indicated with arrows). For a second group of vehicle and ET-1 infused rats, the animals were allowed to recover for 24 h before sacrifice (lower bars; sample collection indicated with arrows). B–E. Analysis of nascent and mature transcripts of IEGs and hypertrophy markers ANF and BNP mRNA in heart tissue from animals infused with ET-1 or saline according to the protocol in A. B. qRT-PCR analysis of ANF mRNA abundance. Each bar represents the mean ± SEM (n = a minimum of 6 rats for each treatment, ****p < 0.0001, NS; not significant). C. qRT-PCR analysis of nascent ANF mRNA. Each bar represents the mean ± SEM (n = a minimum of 6 rats for each treatment, ***p < 0.001, NS; not significant). D. qRT-PCR analysis of BNP mRNA abundance. Each bar represents the mean ± SEM (n = a minimum of 6 rats for each treatment). E. qRT-PCR analysis of nascent BNP mRNA abundance. Each bar represents the mean ± SEM (n = a minimum of 6 rats for each treatment) F. qRT-PCR analysis of *c*-*Fos* mRNA abundance. Each bar represents the mean ± SEM (n = a minimum of 6 rats for each treatment, ****p < 0.0001, NS; not significant) G. qRT-PCR analysis of nascent *c*-*Fos* mRNA abundance. Each bar represents the mean ± SEM (n = a minimum of 6 rats for each treatment, ****p < 0.0001, NS; not significant) Statistical analysis was performed using a one-way ANOVA followed by a post-hoc Holm-Sidak's Multiple Comparison test (symbols above bars/plots).

To test the physiological relevance of our observations, the capacity for a brief exposure of ET-1 to have persistent effects on hypertrophy-associated gene expression was tested *in vivo*. ET-1 was infused in rats *via* the jugular vein over a 15 min time period. A dose of ET-1 was applied that has previously been shown to cause effects on cardiac physiology without substantial changes in blood pressure [25], [26]. Hearts were then either harvested at this point or 24 h later after cessation of the 15 min infusion and recovery of the rats (Fig. 6A). Analysis of gene expression in these hearts was then performed. At the 15 min time point, no change in expression of ANF or BNP was detected, whereas at 24 h, expression of these markers of hypertrophy was observed (Fig. 6B and D). Moreover, the nascent mRNA for these hypertrophic markers was also increased at 24 h, indicating sustained transcription (Fig. 6C and E). Contrastingly, 15 min was sufficient to elicit an increase in expression of *c*-*Fos*, which was sustained, albeit at a significantly lower level until 24 h (Fig. 6F). Nascent transcripts for *c*-*Fos* were also elevated at 24 h, suggestive of continued basal activity of the IEG response (Fig. 6G). These *in vivo* data are consistent with our observations *in vitro* and show relevance of our findings to the pro-hypertrophic effects of ET-1 *in vivo*. While we did not apply an inhibitor of the ET_A_ receptor *in vivo*, at the dose applied, venous levels of ET-1 measured 15 min subsequent to cessation of ET-1 infusion were previously shown to be no different from saline -infused controls [25]. Moreover, ET-1 is cleared from the circulation very rapidly in an ET_B_ receptor dependent manner with a t_1/2_ of 18 s [41]. The effects of ET-1 observed at 24 h are thus not due to sustained elevated levels in the circulation.

### 3.4 MAPK activity downstream of ET-1 is required for induction of hypertrophic gene expression

**Fig. 7:**
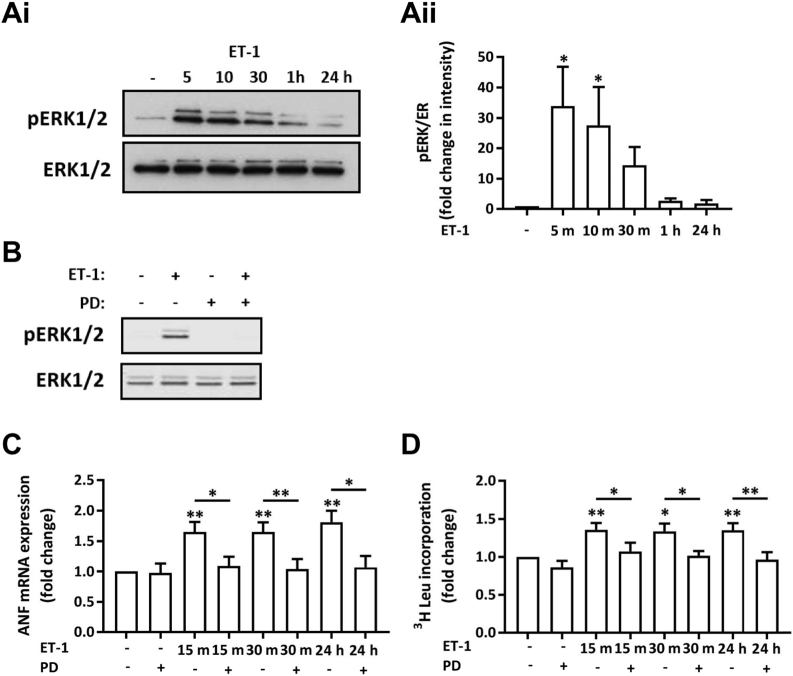
Inhibition of MEK1/2 with PD184352 attenuates the upregulation of hypertrophic markers induced by ET-1. Ai. Representative immunoblot for pERK1/2 and ERK1/2 in a total cell lysate from NRVMs treated with ET-1 for 5, 10, 30, 60 min and 24 h. ii. Quantification of pERK1/2 intensity relative to ERK1/2 in immunoblots as in (Ai) Each bar represents the mean ± SEM (n = 3 primary cell preparations, *p < 0.05). B. Representative immunoblot for pERK1/2 and ERK1/2 following ET-1 application for 15 min ± PD184352. C. qRT-PCR analysis of ANF mRNA abundance relative to the control upon stimulation with ET-1 ± PD. Assays were performed at 24 h. Each bar represents the mean ± SEM (n = 3 primary cell preparations, *p < 0.05, **p < 0.01). D. ^3^H leucine incorporation assay of protein synthesis showing fold change relative to the control upon stimulation with ET-1 ± PD. Each bar represents the mean ± SEM (n = 5 primary cell preparations, *p < 0.05, **p < 0.01). Statistical analysis was performed using a one-way ANOVA followed by post-hoc Holm-Sidak's Multiple Comparison test (symbols above bars/plots).

The data presented above (Fig. 2) indicate that a brief exposure to ET-1 is sufficient to induce hypertrophic indices and IEG expression measured at 24 h despite removal after 15 min and antagonist application. However, measures of hypertrophy were not induced at the point of agonist removal (Supplementary Fig. 3A and B). Given the central role of the ERK1/2 MAPK pathway in the IEG response and pro-hypertrophic action of ET-1 [6], [21], its involvement in the hypertrophic remodelling observed was measured. NRVMs were stimulated with ET-1 for 5, 10, 30, 60 min and 24 h, and levels of active phosphorylated ERK (pERK1/2) assessed. As previously described, a low level of phosphorylated active ERK1/2 (pERK1/2) was detected under baseline conditions [18]. Consistent with previous reports, ET-1 application resulted in a rapid and substantial increase in pERK1/2 levels by 5 min [18], that remained elevated before returning to baseline after the 1 h time point (Fig. 7A). No change in expression of total ERK was detected over this time course. ERK1/2 phosphorylation remained at basal levels at 3 and 12 h suggesting no additional wave of ERK activation (Supplementary Fig. 4). To dissect the time window where the activity of this pathway was required to drive ET-1-induced hypertrophic signalling, ERK activity was inhibited at relevant time points post ET-1 addition. To this end, the direct upstream kinase of ERK1/2, MEK1/2 was inhibited with PD184352 (PD; 10 μM), which is a highly effective in specifically suppressing ERK1/2 activity [42]. Consistent with the necessity of MEK for ERK1/2 activation, PD inhibited the ET-1 stimulated increase in pERK1/2 in NRVMs (Fig. 7B). PD application at 15 or 30 min subsequent to addition of ET-1 prevented the upregulation of ANF mRNA and protein synthesis observed at 24 h (Fig. 7C and D, respectively). These data indicate that ERK1/2 activity beyond the 30 min exposure to ET-1 is necessary for hypertrophy induction at 24 h. Together, these data, generated in experiments in which inhibitors are applied at 15 or 30 min after ET-1 (Act D inhibition of transcription, the presence of nascent IEG and ANF nascent transcripts and PD inhibition of MAPK signalling), strongly support the conclusion that the ET-1-liganded ET_A_ receptor elicits persistent signals (beyond duration of ET-1 exposure) that are required for the hypertrophic response at 24 h.

### 3.5 Inhibition of endocytosis mechanisms potentiates induction of markers of hypertrophy induced by brief exposure to ET-1

**Fig. 8:**
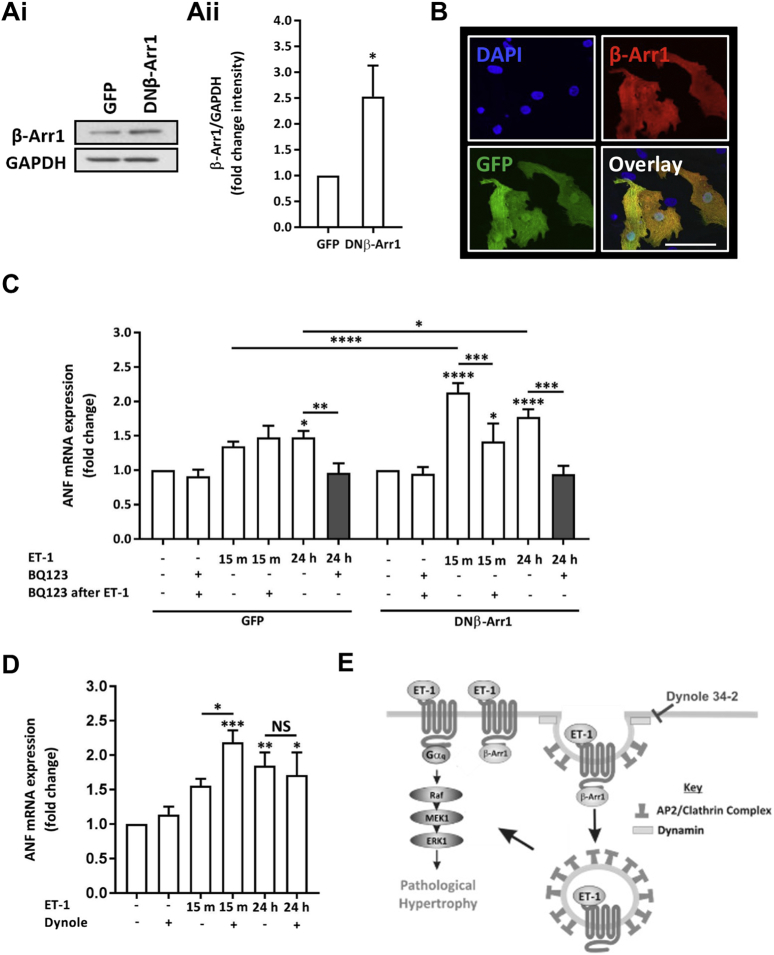
Overexpression of DN β-Arr1 in NRVMs potentiates the induction of ANF mRNA by transient ET-1 exposure and sensitises the response to antagonist. Ai. Representative immunoblot for β-Arr1 in NRVMs infected with either GFP or DN β-Arr1 adenovirus. GAPDH is also shown as a loading control. ii. Quantification of immunoblots for β-Arr1 intensity relative to GAPDH intensity in GFP or DN β-Arr1 adenovirus infected NRVMs. Each bar represents the mean ± SEM (n = 3 primary cell preparations, *p < 0.05). B. Representative confocal images of NRVMs infected with DN β-Arr1 adenovirus. GFP fluorescence is shown in green, β-Arr1 immunostaining in red and nuclei in blue (DAPI). Scale bar represents 50 μm C. qRT-PCR analysis of ANF mRNA abundance relative to the control upon stimulation with ET-1 ± BQ123 in NRVMs infected with GFP or DN β-Arr1 adenovirus. Assays were performed at 24 h. Each bar represents the mean ± SEM (n = 3 primary cell preparations, *p < 0.05, **p < 0.01, ***p < 0.001, ****p < 0.0001, NS; not significant). D. qRT-PCR analysis of ANF mRNA abundance relative to the control upon stimulation with ET-1 ± Dynole 34-2 in NRVMs. Assays were performed at 24 h. Each bar represents the mean ± SEM (n = 3 primary cell preparations, *p < 0.05, **p < 0.01, ***p < 0.001, NS; not significant) E. Model of the proposed pro-hypertrophic action of ET-1 and how the effects of ET-1 exposure are sustained by ligand trapping by the receptor through endocytosis. The mechanism of Dynole 34-2 inhibition of endocytosis is also shown in the Cartoon. Statistical analysis was performed using a one-way ANOVA followed by a post-hoc Holm-Sidak's Multiple Comparison test (symbols above bars/plots) or one-sample *t*-test.

The hypertrophic response observed at up to 48 h, following a stimulus lasting only 15 min, insensitive to subsequent ET_A_ receptor antagonist, yet requiring downstream signalling after the period of agonist exposure, is suggestive of a mechanism in which the active ET_A_ receptor becomes insulated from antagonist following ligand binding. Subsequent to ligand association, GPCRs undergo inactivation through phosphorylation or internalisation *via* an arrestin/clathrin-dependent mechanism. Despite internalisation, ligand-bound ET receptors have been reported to continue to signal from an intracellular location [43]. To determine whether ET_A_ receptor endocytosis underlies the insensitivity of ET-1 action to BQ123, endocytosis was inhibited and effects analysed. To this end, complementary approaches involving genetic and a pharmacological intervention of receptor endocytosis was employed. Given that ET_A_ receptor internalisation occurs through an arrestin/clathrin-dependent mechanism, a dominant negative form of β-Arrestin 1 (DN β-Arr1) was overexpressed to suppress this pathway [44]. The DN β-Arr1 employed harbours a V53D mutation, which has an enhanced affinity for clathrin and impaired interaction with GPCRs due to the presence of the mutation in the primary GPCR binding domain [45], [46]. Overexpression of DN β-Arr1 was achieved using adenovirus, which also mediated expression of GFP under a separate promoter, thereby allowing identification of transduced cells. Transduction of NRVMs with the DN β-Arr1 adenovirus for 8 h resulted in expression of DN β-Arr1 and GFP, which were detected by immunoblotting and confocal imaging respectively (Fig. 8A and B). NRVMs transduced with an adenovirus encoding GFP alone exhibited GFP expression but no increase in β-Arr1 immunoreactivity. DN β-Arr1 expression was detected in the majority of NRVMs transduced with the DN β-Arr1 adenovirus and coincided with GFP expression from the same virus (Fig. 8B). Immunoblot analysis of lysates prepared from DN β-Arr1 transduced cells revealed a significant increase in β-Arr1 expression relative to GAPDH (Fig. 8A). The effect of DN β-Arr1 expression on ET-1-stimulated increase in ANF mRNA was next determined. In these analyses, NRVMs were infected with the DN β-Arr1 adenovirus 24 h in advance of agonist application. NRVMs were stimulated for 15 min with ET-1 after which the agonist was washed off and replaced with BQ123. NRVMs were harvested 24 h later. ANF mRNA abundance (measured at 24 h) was increased following ET-1 application for 15 min in NRVMs infected with control GFP and DN β-Arr1 adenoviruses (Fig. 8C). The magnitude of the increase in ANF mRNA was however significantly greater in myocytes transduced with DN β-Arr1 adenovirus compared to myocytes transduced with the GFP control virus. More significantly, the presence of DN β-Arr1 resulted in acquisition of sensitivity of the ET-1 induced increase in ANF mRNA to BQ123. Specifically, the ET-1 stimulated increase in ANF mRNA at 24 h was significantly blunted in DN β-Arr1 transduced cells in which ET-1 was applied for 15 min and then replaced after 15 min with BQ123. Consistent with that observed for non-infected NRVMs, BQ1213 did not suppress the ET-1 stimulated induction of ANF mRNA in myocytes transduced with GFP adenovirus (Fig. 8C).

The role of receptor endocytosis in ET-1 stimulated hypertrophy was examined further by inhibition of the GTPase activity of dynamin II with Dynole 34-2 (10 μM) [47]. Dynamin is a GTPase, which plays a central role in clathrin-mediated endocytosis, a process involved in the desensitisation of plasma membrane receptors, including ET_A_ receptors [44], [48] in many cells types, including myocytes [49]. Dynole 34-2 was applied to NRVMs following 15 min stimulation with ET-1. Consistent with results obtained by DN β-Arr1 expressing myocytes, Dynole 34-2 addition following exposure to ET-1 for 15 min rendered the ET-1 stimulated increase in ANF mRNA sensitive to BQ123 as well as augmenting the ET-1 stimulated increase in ANF mRNA (Fig. 8D).

The potentiation of the ANF response to ET-1 and its endowment with BQ123 sensitivity supports a model in which through preventing endocytosis, DN-β-Arr1 expression or dynamin inhibition serves to maintain ET_A_ receptor at the sarcolemma, where its activation by ET-1 is enhanced and its inhibition by BQ123 increased (Fig. 8E).

Taken together with prior evidence from the literature, the data presented above suggest a model by which ET-1 promotes ET_A_ receptor activation of downstream signalling and receptor endocytosis [44], [48], [50], [51]. From this intracellular location, liganded ET_A_ receptor signalling persists through a ligand trapping mechanism to sustain the effects of agonist exposure.

## 4 Discussion

Our findings provide new insights into the mechanism by which ET-1 signals *via* its receptor to induce hypertrophic gene expression. Our results show that the pro-hypertrophic effects of ET-1 are engaged within minutes, but endure for longer than 48 h in a manner insensitive to ET-1 removal and ET_A_ receptor antagonism. Notably, the persistent effect of ET-1 was conserved *in vivo*. This property of ET-1/ET_A_ receptor signalling contrasted with signalling from the α1 adrenergic receptor, which was entirely sensitive to antagonist. The persistent hypertrophic effects of ET-1 required the sustained activity of the MAPK pathway downstream of the ET_A_ receptor. Inhibition of classical receptor endocytic pathways rendered myocytes transiently exposed to ET-1 sensitive to ET_A_ receptor antagonism. This suggested that the sustained and antagonist-insensitive action of ET-1 involves a mechanism in which following ET-1 association, the ET-1/ET_A_ receptor complexes is internalised and continues to signal due to insulation from receptor antagonism.

GPCR signalling evokes both acute and long-term effects in the heart as adaptive responses to increased workload/stress. Acutely, changes in physiology are observed that mediate enhanced cardiac function, whereas chronically, a hypertrophic growth response of the myocytes of the heart occurs. When associated with prolonged stress, hypertrophic cardiac growth predisposes to cardiac decompensation and failure [3]. However, the initial stages of the hypertrophic response are detectable within hours [21]. This apparent sensitivity of the heart to the demands of the organism must also be balanced by mechanisms to ensure that the hypertrophic response and the deleterious effects of cardiac function associated with it are not erroneously activated by acute stressors, for example during periods of physiologically-increased sympathetic innervation [52]. As GPCR signalling is key to pathological cardiac remodelling [6], we analysed how the activity of a key downstream pathway – the ERK1/2 MAPK cascade - is coupled to the hypertrophic response induced by ET-1. ET-1 is a potent pro-hypertrophic agonist that elicits acute effects on cardiac physiology as well as longer term effects by inducing pathological cardiac hypertrophic growth [10], [21], [53], [54]. These actions of ET-1 have been extensively modelled *in vitro* in primary cultures of cardiac myocytes. This model has proven a highly tractable and physiologically relevant test-bed to delineate signalling pathways and their contribution to cellular remodelling downstream of the ET_A_ receptor [10], [21], [33]. Despite being necessary and sufficient for the induction of pathological remodelling of the heart *in vivo*, the role of ERK1/2 in cardiac hypertrophy is complicated. In particular, while ERK is required to prevent eccentric cardiac growth, an increase in its activity induces pathological growth [55], [56], [57]. Numerous studies have also demonstrated the requirement for MAPK signalling (ERK1/2) in regulating hypertrophic remodelling *in vitro* [6], [7], [10]. The involvement of ERK1/2 signalling in the transcriptional responses underlying the pro-hypertrophic effects of ET-1 was recently demonstrated by the substantial effects of MAPK inhibition upon ET-1 stimulated changes in the neonatal myocyte transcriptome. These studies revealed that the expression of the majority of genes upregulated in ET-1 stimulated myocytes was dependent on the ERK1/2 cascade [15]. Consistent with these analyses, we observed a potent effect on myocyte hypertrophy and the foetal gene expression programme in myocytes exposed to ET-1 for 24 h. Surprisingly however, ET-1 exhibited similar effects on indices of myocyte hypertrophy when applied for only 30 min and replaced after this initial exposure with ET_A_ receptor antagonist. These effects contrasted with that of the α1 adrenergic receptor agonist PE, which induced expression of markers of hypertrophy that were sensitive to agonist wash off and receptor antagonism (with prazosin). A similar antagonism of PE effects on hypertrophic remodelling in NRVMs has recently been described. However, while in the work presented here prazosin prevented a hypertrophic response before it had developed, in the study described, it reversed hypertrophy that had developed over 24 h [35]. While the ability of ET-1 to elicit such long lasting effects is surprising, the sustained action of ET-1, together with its high affinity for its receptor, contributes to its potency as a pro-hypertrophic agonist and provides a mechanism by which it can act at nanomolar levels in the circulation. In addition to being synthesised by endothelial cells of the vasculature, ET-1 is also produced by cardiac myocytes allowing it to act in an autocrine/paracrine manner [10], [58]. Indeed, ET-1 is elevated in the myocardium during disease and increased *in vitro* in response to pro-hypertrophic stimulation [8], [58].

To explain the long lasting effects of ET-1, we first considered the possibility that the GPCR-proximal downstream signals set in motion a feed forward mechanism by which the initial products of signalling drive subsequent pro-hypertrophic mediators, in a manner independent of the presence of the initiating stimulus. In this way, the cell retains a memory of its exposure to agonist. We considered that this ‘memory’ was achieved through either, or both sustained alteration in signalling or reprogramming of cell transcription [59], [60]. These mechanisms may also determine the nature of reversibility of the response downstream of the initiating signal. These effects are exemplified by the ERK/MAPK signalling pathway, in which differential activation of ERK cascades, either sustained or transient, generate distinct cellular outcomes [61]. Moreover, and in support of this idea, ET-1 stimulates the expression of temporally distinct functional classes of genes in NRVMs [21]. For example, IEG are first apparent, followed by signalling and then structural genes. The requirement for IEG expression and activity for later transcriptional events has been described [62]. Consistent with this model, expression of the IEG *c*-*Fos* was rapidly induced following ET-1 stimulation (< 15 min), while expression of classical markers of hypertrophy developed later. Demonstrating the physiological importance of our findings, these effects of ET-1 observed in the NRVM model were conserved in an *in vivo* model in which ET-1 was infused *via* the jugular vein. Following their induction, despite only a 30 min exposure of cells to agonist, levels of hypertrophy-associated targets remained elevated for 48 h, and surprisingly at a level similar to that observed in cells exposed to agonist for the full duration of the experiment. Through combining the inhibition of transcriptional elongation and analysis of nascent RNA transcripts, we determined that the expression of these hypertrophy-associated genes (the foetal gene programme), measured at 24–48 h and elicited by a brief pulse of ET-1, was due to sustained active transcription and not to carry over of transcripts from this initial period of agonist exposure.

The persistent active transcription, in particular of *c*-*Fos*, a marker of the IEG response, suggested that signalling from the ET_A_ receptor was also elevated for the duration of the time period studied. The temporal profile of ERK1/2 activation following ET-1 stimulation of NRVMs described here and previously did not support this notion [15], [16], [18]. Consistent with previous studies, ERK1/2 phosphorylation is rapidly increased within minutes and then diminishes to baseline 1 h post-agonist stimulation [15]. This time course of signalling mediator activation following GPCR stimulation is widely acknowledged and contributes to avoiding toxicity [7], [10]. To avoid persistent activation, GPCRs and their downstream signalling cascades are configured for regulation at multiple levels. In particular, GPCRs exhibit desensitisation through phosphorylation or endocytosis and signalling pathways induce activity or expression of negative regulators [46], [63]. Indeed, β2 adrenergic receptor signalling only lasts for minutes, with receptors being inactivated by phosphorylation by β adrenergic receptor kinases [64]. Downstream signalling pathways are also subject to strict regulation. For example, the ERK1/2 cascade is regulated upstream through modulation of MEK1 activity, and directly, by DUSP6 *via* dephosphorylation at Thr202 and Tyr204 residues [65]. Knockout of DUSP6 results in increased basal ERK1/2 signalling in the heart and protection against pathology [57]. DUSP1 (also known as MAPK phosphatase 1, MKP1) is rapidly upregulated in response to ET-1 in NRVMs [39] and when overexpressed, inhibits ET-1 stimulated hypertrophic response [66].

Although signalling pathway activation peaks temporally proximal to receptor engagement, results from experiments in which ERK1/2 were inhibited after this initial ERK activation period indicated that signalling beyond this point was required to induce indices of hypertrophy measured at 24 h. These findings would indicate that despite ERK1/2 activation peaking and then decaying to near baseline after 1 h of agonist exposure, a remaining activity, indistinguishable from baseline at time points between 1 and 24 h, must be sufficient to generate the hypertrophic response at up to 48 h. This description and dissection of the window of signalling activity involved in activation of hypertrophic gene expression has not previously been reported, yet has been considered likely important [22]. Given that our analysis of ERK1/2 activity was performed by immunoblotting of lysates prepared from cell populations, dysynchronous low frequency oscillations in signalling activity known to occur downstream of GPCR activation, would not be detected.

The kinetics of signalling from ET_A_ receptors appears more complex than for other GPCRs, for example the α1 adrenergic receptor studied here, which as previously shown is sensitive to antagonist application after agonist [35]. Some of these properties of the ET_A_ receptor may depend upon the cell type/tissue in which the receptor is expressed. Indeed, while ET receptors rapidly undergo ligand-induced desensitisation when overexpressed in Xenopus oocytes, they remain active for a longer time courses in other systems. This is highlighted in the vasculature where ET_A_- mediated elevation of [Ca^2 +^]_i_ in smooth muscle cells is responsible for the prolonged vasoconstriction that occurs subsequent to an initial ET-1 dependent vasodilation due to signalling from the ET_B_ receptor [67]. These chronic actions of ET_A_ receptor signalling reflect our observations in cardiac myocytes. While ET_B_ receptors are expressed in cardiac myocytes [68], all of the effects of ET-1 observed in this study were sensitive to prior application of the ET_A_ receptor antagonist BQ123. Despite its high affinity for the ET_A_ receptor [69] however, BQ123 did not affect ET-1 receptor signalling when applied subsequent to agonist. This inability of BQ123 to antagonise the effect of ET-1 may be due to the apparent irreversible and ‘covalent’ binding of ET-1 to its receptor [50]. Alternatively, the lack of an effect of BQ123 can also arise through a mechanism in which ligand bound ET_A_ receptors internalise from the sarcolemma where they are then insulated from extracellular BQ123 but continue to signal. Indeed, imaging of GFP-tagged ET_A_ receptors, monitoring of radioisotope labelled ET-1 and immunofluorescent studies have revealed that ET-1/ET_A_ receptor complexes are internalised into caveolin-containing vesicles within 10 min of ET-1 application [43], [44]. Intriguingly, following internalisation, the ET-1/ET_A_ receptor complex continues to signal providing a mechanism for this long lasting effect of ET-1 [44]. In contrast to other GPCRs, including the ET_B_ receptor, ET_A_ receptors are not inactivated by phosphorylation thereby allowing persistent signalling [51], although this feature of ET_A_ receptor regulation is debated [44]. The possibility for receptors to continue to signal following clathrin-mediated endocytosis is not restricted to ET_A_ receptors and has described for the EGFR and also α1 adrenergic receptors and has been termed ligand trapping [35]. However, the activity of intracellular α1 adrenergic receptors after internalisation has been reported to be inhibited by prazosin, which due to its hydrophilic properties is able to access these intracellular receptors [35]. Recent studies have also suggested the existence of intracellular ET receptors that respond to ‘intracrine’ ET-1 [58]. The mechanism by which this is brought about remains to be fully established.

Receptor endocytosis often involves β-Arr recruitment to the active receptor, which then initiates clathrin-mediated endocytosis. Here, we found that expression of a DN β-Arr1 to prevent ET_A_ receptor endocytosis led to a potentiation of the effect of ET-1 on ANF expression. Moreover and perhaps surprisingly, this manoeuvre resulted in the acquisition of sensitivity of the ET-1 stimulated increase in indices of hypertrophy to BQ123. Together with similar observation obtained using pharmacological inhibition of dynamin to inhibit endocytosis, these data would suggest that β-Arr mediated internalisation underlies the mechanism that allows ET-1 signalling to persist. Notably, the acquired ability of BQ123 to suppress the effects of ET-1 when ET_A_ receptors are forced to remain on the cell surface would also suggest that interaction of ET-1 with its receptor is not irreversible. The maintenance of high level transcription of markers of hypertrophy in the face of endocytosis was surprising given that receptor endocytosis is often a pathway for lysosomal degradation. As indicated above, internalised ET_A_ receptors can continue to signal [43], and have been reported to be recycled to the plasma membrane *via* a pericentriolar recycling compartment rather than directed to the lysosomes for degradation [44], [48].

While our results show continued signalling from ET-1/ET_A_ receptors for up to 48 h, our *in vitro* experimental model did not allow us to probe longer-term effects. Indeed, in animals, although initial makers of hypertrophy are detectable within hours, hypertrophy endpoints develop over a longer time course and future studies will explore how these develop subsequent to transient hypertrophic stressors [20], [22]. It is clear however that, upon removal of a pro-hypertrophic stimulus, the heart does not return to its pre-stimulated state for a number of days suggesting some persistent signalling and long term effects of ET-1 upon cardiac physiology are also observed *in vivo* [58]. For example, continuous perfusion of ET-1 *via* osmotic minipump results in a loss of ET-1 induction of inotropy after 2 weeks [70]. How ET-1/ET_A_ receptor signalling is eventually inactivated remains to be determined although ET-1 levels are rapidly cleared *in vivo* by ET_B_ receptors [41]. Indeed, venous levels of ET-1 following infusion of the amount of ET-1 used in this study returns to baseline within 15 min [26]. ET_A_ as well as ET_B_ receptors also desensitise *via* palmitoylation [71], [72]. This mechanism requires further study as it is unclear how this may affect its role in the initiation and termination of hypertrophic signalling [72].

Intriguingly, despite desensitisation and the persistent nature of the ET-1 response, the ET-1 signalling system is amplified in cardiovascular disease including in the cardiac myocyte compartment. Under stress conditions such as stretch and β adrenergic stimulation *in vitro*, or increased haemodynamic load *in vivo*, the expression of ET-1, the endothelin converting enzyme (ECE) and ET_A_ receptors is increased [73]. This augmentation of the ET-1 pathway may thus serve to maintain cellular sensitivity to ET-1 and to accommodate for the eventual degradation of internalised receptors.

## 5 Conclusions and implications

ET-1 has been shown to drive progression of CVD and HF. Since the discovery of ET-1 and its cognate receptors, there has been a focus on identifying specific inhibitors for potential therapeutic use. However, results of specific ET_A_ receptor antagonists in clinical trials have been disappointing [74]. As well as the high affinity interaction between ET-1 and ET_A_ receptor, the multifaceted role of ET-1 in regulating the cardiovascular system can make ET receptors an unpredictable therapeutic target [75]. Lack of efficacy of antagonists upon the ET_A_ receptor may in part be explained by the continued signalling of ET receptors from the intracellular space. Our findings that inhibitors of endocytosis render ET_A_ receptors sensitive to antagonists may provide an avenue for improved therapy at this receptor. There is currently a limited understanding of ET-1/ET_A_ receptor interaction during the induction of cardiac hypertrophy. The finding that ET-1/ET_A_ receptor signalling persists in NRVMs and *in vivo* has important implications for the mechanisms underlying the prolonged duration of ET-1-induced hypertrophic gene transcription. It is clear from these data that active transcription is maintained even following removal of a stimulus. In the *in vivo* situation, the persistence of hypertrophic transcription would serve to maintain the pathological remodelling of the heart in the absence of continued stimulus. Indeed, *in vivo* debanding models have revealed that hypertrophy can take up to 4–8 weeks to reverse following removal of the pressure overload [76], [77], [78]. Further study of ET_A_ receptor pharmacology, the dynamics of intracellular signalling pathways and desensitisation mechanisms are thus required to gain insight into how this receptor can be targeted for therapeutic benefit.

## Declaration of interest

The authors report no conflicts of interest in this work.

## Funding sources

This work was supported by an Odysseus Award from the Research Foundation Flanders (FWO, 90663), the Babraham Institute, the Biotechnology and Biological Sciences Research Council (BBSRC, BBS/E/B/0000C116, BBS/E/B/000C0402) and The Royal Society (University Research Fellowship to HLR, UF041311). CRA and FMD were supported by studentships from the BBSRC (BBS/E/B/0000L726 and BBS/E/B/0000L715 respectively).

## Author contributions

HLR, CRA, ELR and FMD conceived and designed the experiments, CRA, ELR and HLR analysed and interpreted the data, CRA, ELR and FMD performed the experiments, HLR and CRA drafted the manuscript, which was critically revised by HLR and FMD.

## Appendix A Supplementary data

Supplementary material Image 1
