# Supplementary material for: Endothelin-1 promotes hypertrophic remodelling of cardiac myocytes by activating sustained signalling and transcription downstream of endothelin type A receptors
Source: Cell Signal. 2017 Aug;36:240–54. doi: 10.1016/j.cellsig.2017.04.010 (PMC5486433; doi:10.1016/j.cellsig.2017.04.010)
Supplement: Supplementary file 1 — Supplementary material [file mmc1.docx]

**Supplementary Materials**

| **Marker Type** | **Gene** | **Primer Sequence (5’-3’)** | **Primer**  **Concentration (nM)** |
| --- | --- | --- | --- |
| Housekeeping Genes | *Gapdh* | F- CAAGATGGTGAAGGTCGGTGT  R- RGGTCGTTGATGGCAACAATG | 200  300 |
|  | *TnnT2* | F- GGAAGAGGAAGACTGGAGCGA R- CTCACCATCCTCCTCCTCCAC | 200  300 |
|  | *B2m* | F- CCGAGACCGATGTATATATGCTTG R- AACTGGTCCAGATGATTCAGAGC | 300  300 |
|  | *Ywhaz* | F- ACCCACTCCGGACACAGAAT  R- AGGCTGCCATGTCATCGTA | 200  200 |
|  | *Tata BP* | F- CGTACATCTCAGCTGCTTCC  R- GTTATCGTCACGCACCATGA | 200  200 |
| Hypertrophic Markers | *Nppa* | F- CGTATACAGTGCGGTGTCCAAC  R- CATCTTCTCCTCCAGGTGGTCTAG | 200  200 |
|  | *Nppb* | F- AAGTCCTAGCCAGTCTCCAGAACA R- TTGAGAGCTGTCTCTGAGCCATT | 300  300 |
| Hypertrophic Marker: Nascent Transcript | *Nppa* | F- CTGATGGGTGTCCCTTGAGT  R- CTGGCCTCTTCGGTAAACTG | 200  300 |
|  | *Nppb* | F- TCAGGAAACGGAAAGATTGG  R- AGACCTCAGACACACACACACA | 300  100 |
|  | *c-Fos* | F- TGCTTCTCTCTCCCTGCTTC  R- TGTCACCGTGGGGATAAAGT | 200  200 |
| IEG | *c-Fos* | F- CCGACTCCTTCTCCAGCATG  R- GTGGAGATGGCTGTCACCGT | 200  300 |

#### Table 1: Housekeeper and hypertrophic marker primer sequences and concentrations for qRT-PCR.

**
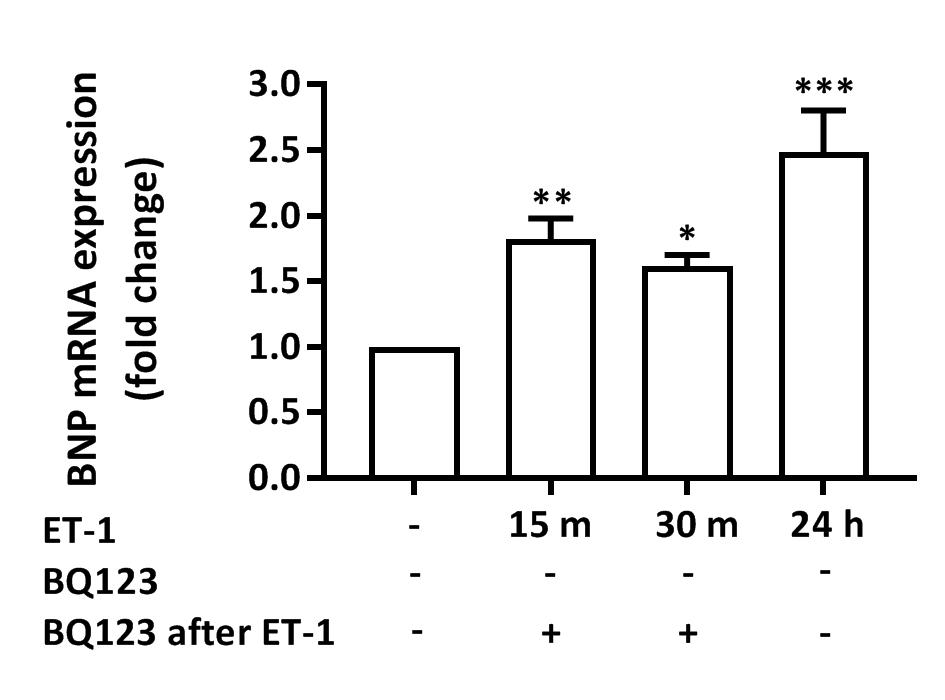
**

**Supplementary Fig 1. Stimulation of NRVMs with ET-1 for 15 min or 24 h induced upregulation of BNP.**

qRT-PCR showing fold change in BNP mRNA expression relative to the control upon stimulation with ET-1 (100 nM) ± BQ123 (1 µM). Assay performed at 24 hours. Each bar represents the mean ± SEM (n=3 primary cell preparations, *p < 0.05, **p < 0.01 and *** p <0.001). Statistical analysis performed using one-way ANOVA with Holm-Sidak’s multiple comparisons test (symbols above bars/plots).


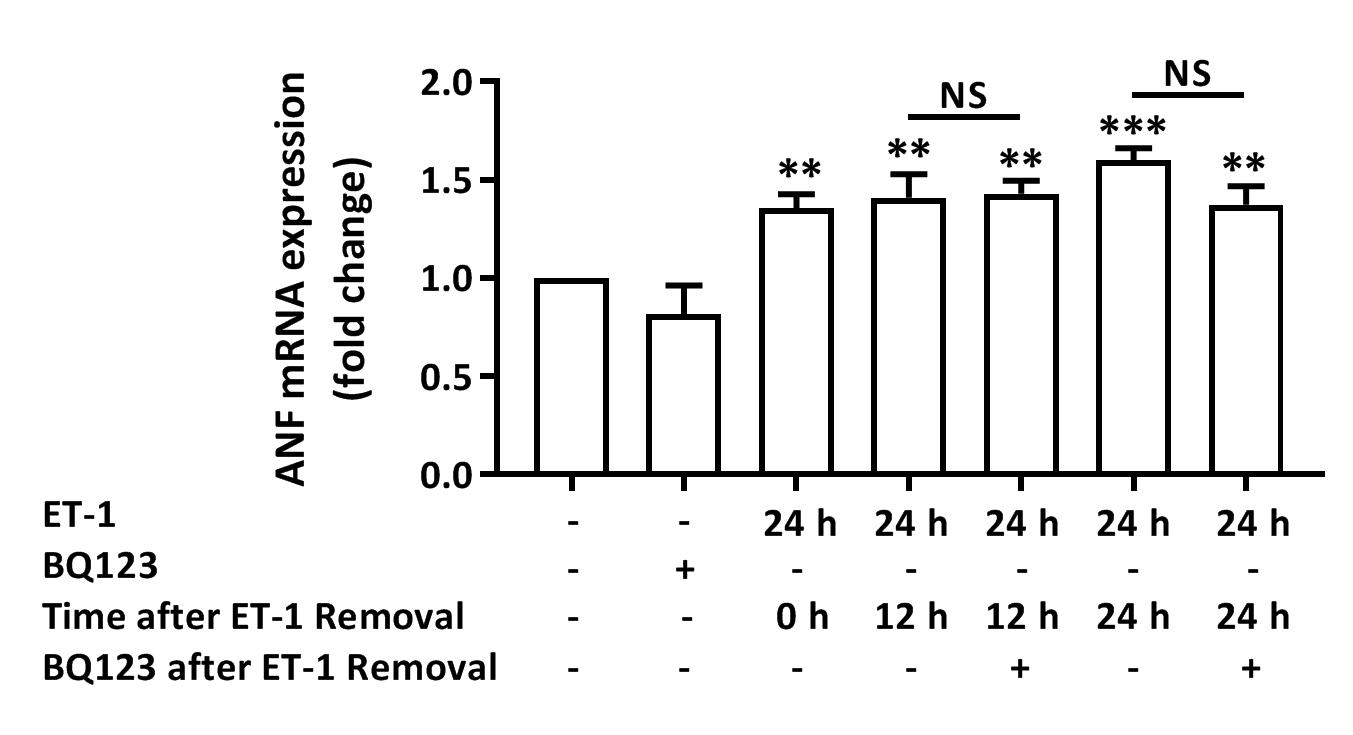


**Supplementary Fig 2. Prolonged ET-1 exposure followed by ET_A_ receptor antagonist addition, induced upregulation of hypertrophic markers in NRVMs over an extended timecourse.**

qRT-PCR analysis of ANF mRNA abundance relative to the control upon stimulation with ET-1 (100 nM) for 24 hours ± BQ123 (1 µM) at 0, 12 or 24 hours. Each bar represents the mean ± SEM (n=3 primary cell preparations, **p<0.01, ***p<0.001, NS; not significant). Statistical analysis performed using one-way ANOVA with Holm-Sidak’s multiple comparisons test (symbols above bars/plots).


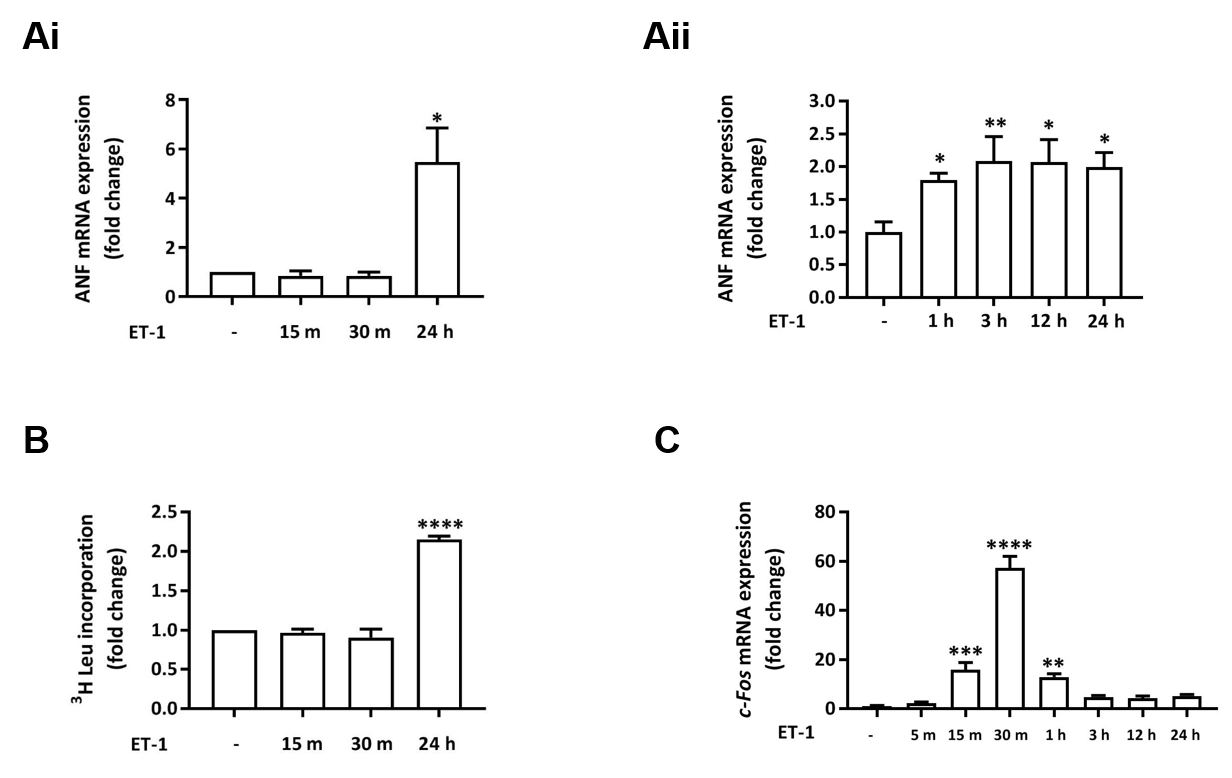


**Supplementary Fig 3. Stimulation of NRVMs with ET-1 for 15 or 30 minutes upregulates immediate early gene response in the absence of hypertrophic remodelling.**

**Ai.** qRT-PCR analysis of ANF mRNA abundance relative to control 15, 30 minutes or 24 hours immediately following ET-1 (100 nM) application. Each bar represents the mean ± SEM (n=3 primary cell preparations, *p < 0.05). **Aii.** qRT-PCR extended protocol analysis of ANF mRNA abundance relative to control 1, 3, 12 or 24 hours immediately following ET-1 (100 nM application. Each bar represents the mean ± SEM (n=4 primary cell preparations, *p < 0.05, **p < 0.01). **B.** Fold change in total protein synthesis 15, 30 minutes or 24 hours immediately following ET-1 (100 nM) application. Each bar represents the mean ± SEM (n=3 primary cell preparations, ****p < 0.0001). **C.** qRT-PCR analysis of *c-Fos* mRNA expression relative to the control, immediately following stimulation with ET-1 (100 nM) for 5, 15, 30 minutes, 1, 3, 12 or 24 hours. Each bar represents the mean ± SEM (n=3 primary cell preparations, **p < 0.01, ***p < 0.001, ****p < 0.0001). Statistical analysis performed using one-way ANOVA with Holm-Sidak’s multiple comparisons test (symbols above bars/plots).


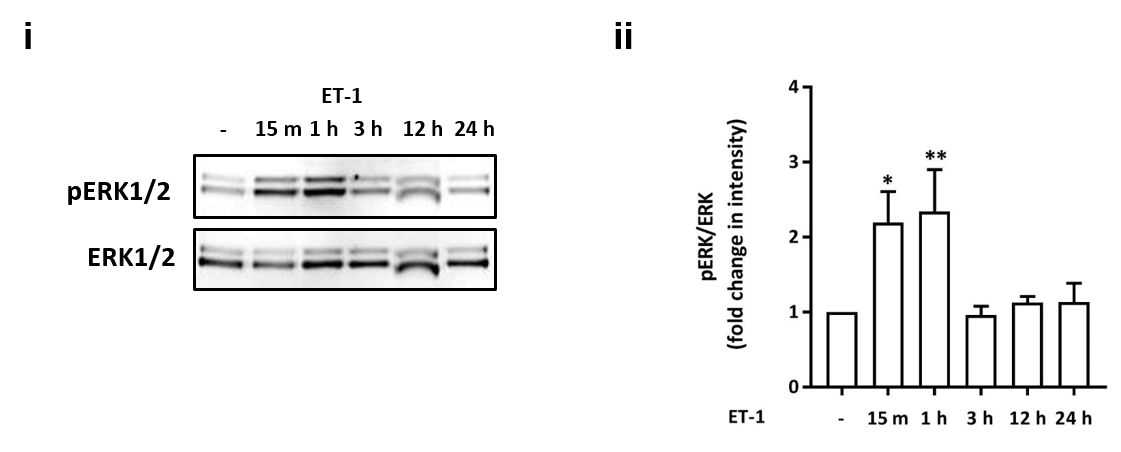


**Supplementary Fig4. ERK phosphorylation following exposure to ET-1 rapidly peaks then declines to baseline after 1 hour. i.** Representative immunoblot for pERK1/2 and ERK1/2 in a total cell lysate from NRVMs treated with ET-1 (100 nM) for 15 minutes, 1, 3, 12 and 24 hours. **ii.** Quantification of pERK1/2 intensity relative to ERK1/2 in immunoblots as in **(Ai)** Each bar represents the mean ± SEM (n=4 primary cell preparations, *p < 0.05, **p < 0.01).
